# Temporal Variabilities in Genetic Patterns and Antibiotic Resistance Profiles of Enterococci Isolated from Human Feces

**DOI:** 10.1264/jsme2.ME15158

**Published:** 2016-06-03

**Authors:** Masateru Nishiyama, Hidetaka Shimauchi, Yoshihiro Suzuki

**Affiliations:** 1 Department of Environment and Resource Science, Interdisciplinary Graduate School of Agriculture and Engineering, University of MiyazakiGakuen Kibanadai-Nishi 1–1, Miyazaki 889–2192Japan; 2 Department of Civil and Environmental Engineering, Faculty of Engineering, University of MiyazakiGakuen Kibanadai-Nishi 1–1, Miyazaki 889–2192Japan

**Keywords:** temporal variability, enterococci, human feces, genetic pattern, antibiotic resistance profile

## Abstract

Temporal variabilities in the genetic patterns and antibiotic resistance profiles of enterococci were monitored over a 7-month period. *Enterococcus faecalis* isolates (103 strains) collected from feces showed only one genetic pattern and antibiotic resistance profile within 0 d and 30 d. In contrast, after 60 d and 90 d, the genetic patterns and antibiotic resistance profiles of all *E. faecalis* isolates (8 strains) clearly differed within 30 d. These results indicate that the genetic patterns and antibiotic resistance profiles of *E. faecalis* in human feces changed to completely dissimilar patterns between 1 and 2 months.

The composition of the microbiota in human and animal gastrointestinal tracts exhibits age- and diet-related changes and has been associated with the health of and diseases in the host (2, 11). Antibiotic treatments for illnesses affect not only the target microorganisms, but also host-associated microbial communities, particularly those in the intestine (15). Therefore, the gut microbiota of the host varies markedly depending on multiple factors. Consequently, genetic patterns within a single species level may also vary in the gastrointestinal tract.

Enterococci are Gram-positive lactic acid bacteria that form a part of the intestinal microflora in human and animal gastrointestinal tracts. Due to their ubiquity in human and warm-blooded animal feces and their long-term survival in the environment, enterococci have been adopted as indicators of fecal contamination in aquatic environments (10, 14). Conversely, some enterococci occasionally cause opportunistic infections in compromised hosts and aggravate nosocomial infections (7). Enterococci are intrinsically resistant to a broad range of antimicrobial agents; they have become resistant to some antibiotics through the acquisition of antibiotic resistance genes located on plasmids or transposons from other organisms (4, 16). Vancomycin-resistant enterococci (VRE) are the most problematic bacteria causing nosocomial infections (16). Since nosocomial infections caused by VRE and antibiotic-resistant bacteria have been identified as the route of transmission, pulsed-field gel electrophoresis (PFGE) has been employed to compare genotypic characteristics within the same species. In addition, a PFGE analysis has been applied to track the source of fecal contamination in aquatic environments, including polluted river water and coastal areas (5, 6). Therefore, the information derived from the predominant *Enterococcus* species, temporal variability in species, and the conservation of genotypes in the human gut is crucially important not only for identifying the route of nosocomial infection, but also for investigating fecal pollution in aquatic environments.

In the present study, the succession of the intestinal bacterial community in human feces was investigated for a 7-month period. Variations in the predominant species of *Enterococcus* in human feces were monitored. The conservation of the genetic pattern of *Enterococcus* species in feces was confirmed, and research on the relationship between the genotypes of enterococci and antibiotic resistance profiles was conducted.

## Materials and Methods

Fresh fecal samples were obtained from a healthy adult male volunteer between the age of 20 and 30 years. The volunteer received no antibiotics for 1 month prior to the start of the study until the end of the experiment. Prior to the long-term monitoring experiment, five fecal subsamples were collected from identical feces in order to analyze homogenous bacterial density in feces. In the long-term monitoring experiment, feces were collected on 0 d, 7 d, 14 d, 30 d, 60 d, 90 d, 180 d and 210 d. The fecal sample was scraped using a cotton bud. The cotton bud was placed in a sterile tube, following which 10–40 mg of the fecal sample was subsequently suspended in 10 mL of sterile saline and thoroughly vortexed for 3 min (18). Suspended fecal samples were diluted using sterile saline (0.85 w/v %), and tested for total coliforms, *Escherichia coli*, and enterococci. Total coliforms and *E. coli* were analyzed using the Colilert^®^-18 kit (Idexx Laboratories, Westbrook, ME, USA). One milliliter of the suspended fecal sample was diluted with distilled water (total volume: 100 mL). This kit provides a MPN value based on the presence or absence of fluorescence in Quanti-Tray. Total coliform and *E. coli* counts were evaluated using mean values. Enterococci were analyzed using the membrane filtration (MF) method (17). A 1- or 10-μL fecal suspension was diluted with 10 mL sterile distilled water. The samples were then filtered through a sterile membrane filter (0.45-μm pore, 47-mm diameter, mixed cellulose ester; Advantec, Tokyo, Japan). After being incubated, colonies on the filter showing blue halos were regarded as putative enterococci. The number of enterococci using MF was averaged and expressed as CFU g^−1^ of water. All statistical analyses on bacterial counts were performed using Microsoft Excel. Bacterial counts were analyzed by a two-sample *t*-test (*P*<0.05). One hundred single colonies were randomly isolated from the mEI agar plate and streaked on a Todd–Hewitt agar plate (1.5% agar; Bacto; Becton, Dickinson, NJ, USA). All colonies were isolated from plates showing <100 colonies.

*Enterococcus faecium* and *E. faecalis*, which form the main types of enterococci in the human gastrointestinal tract (12, 13), were identified using a PCR analysis. Genomic DNA was extracted using the InstaGene Matrix (Bio-Rad Laboratories, Hercules, CA, USA) according to the instructions of the manufacturer. 16S-rRNA-based PCR identification was performed using Phusion High-Fidelity DNA Polymerase (Thermo Fisher Scientific, Waltham, MA, USA). PCR primer sets and a PCR program have been reported for *E. faecalis* (9) and *E. faecium* (8). *E. faecalis* NBRC 100481 and *E. faecium* NBRC 100486 were used as positive controls in all PCR experiments.

In order to clarify genetic relatedness among enterococci, PFGE was performed using a modified procedure designed by the National Institute of Infectious Diseases, Japan (1). Twenty-seven out of 100 isolates identified as *E. faecalis* on each sampling day were randomly selected in isolates and analyzed using PFGE. When <27 isolates were available, all *E. faecalis* isolates were analyzed. The PFGE analysis was performed according to previously described methods (6). The minimum inhibitory concentration (MIC) of each antibiotic was determined using the agar dilution method according to the Clinical and Laboratory Standards Institute (CLSI) guidelines (3). A reference strain of *E. faecalis* ATCC 29212 was used as a quality control. The antibiotics used in the present study included ampicillin (Wako Pure Chemical Industries, Osaka, Japan), penicillin G (Wako Pure Chemical Industries), chloramphenicol (Sigma-Aldrich), ciprofloxacin (LKT Laboratories, St. Paul, MN, USA), erythromycin (Wako Pure Chemical Industries), high-level gentamycin (Wako Pure Chemical Industries), high-level streptomycin, imipenem (LKT Laboratories, USA), tetracycline (Wako Pure Chemical Industries), and vancomycin (Wako Pure Chemical Industries).

## Results and Discussion

Total coliform and *E. coli* counts in 5 fecal subsamples were the same with concentrations ranging between 7.9×10^6^ and 5.3×10^7^ MPN g^−1^ (avg counts: 3.2×10^7^ MPN g^−1^, *n*=5, Table S1). The number of enterococci in fecal subsamples was lower than those of total coliforms and *E. coli* (avg counts: 1.8×10^6^ CFU g^−1^, *n*=5). Total coliform, *E. coli*, and enterococci counts were similar among five fecal subsamples in feces (Table S1). Thirty colonies per fecal subsample were randomly selected from the agar plates spread with five different fecal samples (a total of 150 colonies). All isolates were identified as *E. faecalis* based on species-specific PCR assays. The abundance of *Enterococcus* species in each fecal subsample was in the same proportion. Therefore, bacterial abundance was constant among the subsamples collected from the same feces.

**Fig. 1:**
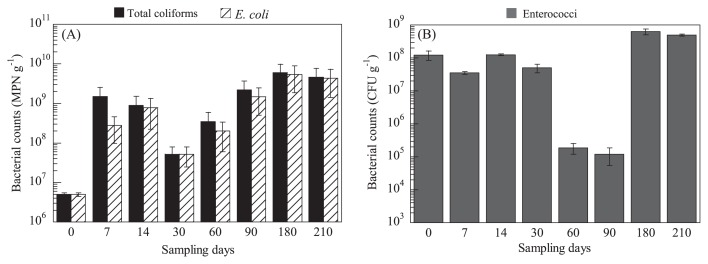
Bacterial counts in feces during the study period. (a) The black and shaded bars show the counts of total coliforms and *Escherichia coli*, respectively. Error bars indicates 95% confidence lines. (b) The grey bar shows the average value of enterococci. Error bars indicate standard deviations, obtained from triplicate experiments.

The number of coliforms in feces in the long-term experiment ranged between 5.0×10^6^ and 6.0×10^9^ MPN g^−1^ (Fig. 1). The density of *E. coli* in fecal samples varied between 5.0×10^6^ and 5.4×10^9^ MPN g^−1^ during the study period. No significant differences were observed in total coliform or *E. coli* counts in each fecal sampling period (t-test, *P*=0.37). Variations in enterococci counts among samples collected on different days were larger than those in coliform counts (coliform coefficient of variation: 1.04, enterococci coefficient of variation: 1.24). Total coliform and *E. coli* counts decreased between 30 d and 60 d, whereas that of enterococci increased. Recent studies have indicated that the gut microbiota varies with many factors, such as diet, food, and disease (10). The consumption of a high-fat and low-fiber or low-fat and high-fiber diet for 10 d has been shown to induce significant changes in the gut microbiota (20). Therefore, the human gut microflora of the volunteer may have markedly changed between 30 d and 60 d; however, the specific factors influencing the population shift were not clarified in the present study.

The proportions of *E. faecalis* and *E. faecium* among the isolated strains are shown in Table 1. The predominant *Enterococcus* species isolated from the initial day (0 d) was *E. faecalis* (91%). The proportion of *E. faecalis* significantly decreased by 60 d (25%), and *E. faecalis* isolates were no longer detected in the 180 d–210 d samples. In contrast, the percentage of *E. faecium* increased until 60 d and became the predominant species. However, 99 isolates in the fecal sample collected on 210 d were not identified as *E. faecalis* or *E. faecium*. In the present study, other *Enterococcus* species were not identified; previous studies reported that minor *Enterococcus* species include *E. avium*, *E. casseliflavus*, *E. durans*, *E. gallinarum*, and *E. raffinosus*, which were previously isolated from the human gut and/or feces (4, 19). The abundance of *Enterococcus* species in the human gut has been suggested to change from major to minor species.

The genomic patterns of *E. faecalis*, which was the predominant species isolated from the fecal samples from the initial day, were tracked by PFGE using a *Sma*I restriction enzyme. The PFGE band patterns (*i.e.* the number and location of each band) of all 27 isolates from the initial day were identical. In 7 d and 14 d isolates, the genetic band patterns of each of the 27 isolates were the same as those on the initial day. This result suggested the presence of *E. faecalis* with the same PFGE pattern (*i.e.* clonal population) in human fecal samples across these sampling dates. In addition, out of the 27 isolates examined, the genetic patterns of 22 isolates were identical to those of the isolates from 0 d–14 d. However, the PFGE patterns of the remaining 5 isolates were clearly different from the genetic patterns isolated on 14 d. The number of *Enterococcus* in feces was significantly lower on 60 d than on 30 d (Fig. 1). After 60 d, the percentage of total enterococcal isolates of *E. faecalis* markedly decreased, and the predominant species changed from *E. faecalis* to *E. faecium* (Table 1). Therefore, *E. faecalis* with a different genotype was colonized by enterococcal succession in the human gut and *E. faecalis* of the major PFGE type (type A) decreased, whereas the minor PFGE genotype became predominant. Source tracking relevant to nosocomial infections and aquatic environments has been applied based on a DNA fingerprinting method, which has the ability to distinguish different infected bacteria within a species based on genotypic characteristics (1, 6, 16). Since the PFGE type in the *Enterococcus* isolates remained in the human intestinal flora, at least until 30 d, source tracking based on the *E. faecalis* genotype using a PFGE analysis may provide useful information.

**Fig. 2:**
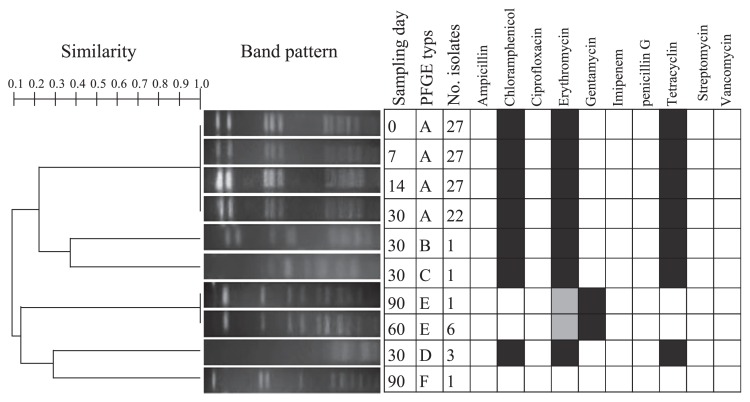
Dendrogram of pulsed-field gel electrophoresis types and antibiotic resistance profiles obtained from *Enterococcus faecalis* isolates. Regarding the antibiotic resistance phenotype, black indicates resistance, grey indicates intermediate resistance, and white indicates susceptibility

As shown in Fig. 2, 103 strains isolated from each sampling day (0, 7, 14, and 30) were classified as having a common genetic pattern (type A) belonging to the same cluster with a 1.0 similarity level (= 100% similarity). A total of 103 isolates showed resistance to chloramphenicol, erythromycin, and tetracycline. Furthermore, all isolates of the same PFGE type showed the same antibiotic resistance profiles. The PFGE types of 60 d and 90 d were classified into the same cluster at a 1.0 similarity level (type E), but differed from type A. All type E isolates (two isolates) exhibited high-level resistance to gentamycin (MIC: >500 μg ml^−1^) and intermediate resistance to erythromycin (MIC: 2 μg mL^−1^). Six isolates collected from 30 d and 90 d were not classified into either type A or type E clusters at a low similarity level (types B, C, D, and F). Although there was only one subject in the present study, the succession of the genetic pattern at the species level was raised by 30 d in the human gut and concurrent with changes in antibiotic resistance by bacteria in the human gut.

## Conclusion

This study investigated temporal variabilities in the genetic patterns and antibiotic resistance profiles of enterococci isolated from human feces. Our results indicate that the genetic patterns and antibiotic resistance profiles of *E. faecalis* in human feces were changed to completely dissimilar patterns between 1 and 2 months. These results support source tracking based on *E. faecalis* genotyping and phenotyping methods.

## Supplementary Material



## Figures and Tables

**Table 1:** Proportion of enterococci in feces during the sampling period.

Sampling day	No. isolates	*E. faecalis*	*E. faecium*	other

		Percentage of isolates (no. isolates)		
0	100	91 (91)	0 (0)	9 (9)
7	100	69 (69)	3 (3)	28 (28)
14	100	47 (47)	14 (14)	39 (39)
30	100	50 (50)	18 (18)	32 (32)
60	24	25 (6)	42 (10)	33 (8)
90	36	6 (2)	6 (2)	88 (32)
180	100	0 (0)	27 (27)	73 (73)
210	100	0 (0)	1 (1)	99 (99)
