# Supplementary material for: Temporal Variabilities in Genetic Patterns and Antibiotic Resistance Profiles of Enterococci Isolated from Human Feces
Source: Microbes Environ. 2016 Jun 3;31(2):182–5. doi: 10.1264/jsme2.ME15158 (PMC4912156; doi:10.1264/jsme2.ME15158)
Supplement: Supplementary file 1 [file 31_182_s1.pdf]

Supplemental material

Table S1 Bacterial density of 5 fecal subsamples in feces.

Fecal subsamples	Total coliform ($\times10^7$ MPN g^{-1})	*E. coli* ($\times10^7$ MPN g^{-1})	Enterococci ($\times10^6$ CFU g^{-1})
No.1	5.3	5.3	2.0
No.2	4.8	4.8	0.83
No.3	1.7	1.7	0.48
No.4	3.2	3.2	6.3
No.5	0.79	0.79	0.37
Mean ± SD	3.2±1.7	3.2±1.7	2.0±2.2
Coefficient of variation	0.54	0.54	1.12
